# Editorial: Pediatric and perinatal cardiology: insights, advances and updates

**DOI:** 10.3389/fped.2026.1780277

**Published:** 2026-02-13

**Authors:** Nathalie Jeanne Magioli Bravo-Valenzuela, Edward Araujo Júnior, Heron Werner Junior

**Affiliations:** 1 Pediatrics Department, Instituto de Puericultura e Pediatria Martagão Gesteira/Federal University of Rio de Janeiro, Rio de Janeiro, Brazil; 2 Department of Pediatrics, Pediatric Cardiology, School of Medicine, Federal University of Rio de Janeiro (UFRJ), Rio de Janeiro, Brazil; 3 Cardiology Institute, University Cardiology Foundation (IC/FUC), Porto Alegre, Brazil; 4 Department of Obstetrics, Paulista School of Medicine, Federal University of São Paulo (EPM-UNIFESP), São Paulo, Brazil; 5 Discipline of Woman Health, Municipal University of São Caetano do Sul (USCS), São Caetano do Sul, Brazil; 6 Department of Fetal Medicine, Biodesign Laboratory DASA/PUC, Rio de Janeiro, Brazil

**Keywords:** arrhythimas, cardiomyopathy, congenital heart disease—cardiac, coronary anomalies, deep learning—artificial intelligence, genetics, kawasaki disease (KD), pediatrics

Pediatric and perinatal cardiology are rapidly evolving fields. Technological innovation, translational research, and an increasing understanding of cardiovascular disease throughout the lifespan have contributed to this evolution. From the initial diagnosis of fetal heart conditions to the long-term follow-up of children with complex heart diseases survivors, contemporary pediatric cardiology necessitates precision, multidisciplinary collaboration, and sustained longitudinal care. The research topic under consideration is entitled “Pediatric and Perinatal Cardiology: Insights, Advances, and Updates in Frontiers in Pediatrics is a collection of original research articles, reviews, and case reports that collectively reflect the journal's stated priorities and highlight emerging directions in clinical practice and scientific investigation.

Considering the significant role played by anomalous origins of coronary arteries in sudden death cases among athletes, it is crucial to emphasize the importance of diagnostic methodologies employed to identify coronary anomalies and evaluate myocardial perfusion. Notably, anomalous origins of the right coronary artery have been identified as a prominent contributing factor to sudden cardiac deaths in younger demographics of athletes. Cardiovascular magnetic resonance with dobutamine stress (DSCMR) is being increasingly utilized in the functional assessment of coronary arteries, however, there is limited data in the pediatric population regarding utilization of this tool. In this setting, Sachdeva et al. (1) conducted a study evaluating myocardial feature tracking during DSCMR in pediatric patients with coronary anomalies and demonstrated how global circumferential strain can objectively identify inducible ischemia and stress-related wall motion abnormalities, even in the presence of preserved systolic function. A particular emphasis is placed on the expanding significance of myocardial deformation imaging and progressive cross-sectional modalities in pediatric cohorts. This study integrates the practical application of myocardial deformation imaging with DSCMR imaging. In this manuscript, the authors demonstrate the feasibility of assessing myocardial deformity at rest and under stress, thereby reinforcing the concept that incipient myocardial dysfunction may precede evident clinical deterioration. The authors further posit that CMR and strain may serve as a useful tool in risk stratification in children with coronary artery anomalies (1).

Interventional strategies represent a further critical focus of this Research Topic. In a comparative analysis of right ventricular outflow tract stenting and modified Blalock-Taussig shunt as palliative therapy in children with cyanotic congenital heart disease, Prakoso et al. (2) demonstrated comparable rates of serious adverse cardiovascular outcomes between the two approaches. The present study lends support to the feasibility of catheter-based palliative care for relief of right ventricle outflow tract stenosis, whilst concomitantly emphasizing the importance of individualized decision-making. Such decision-making ought to be based on patient age, anatomy, institutional experience, and the availability of local resources. Arrhythmia management represents another area of significant progress reflected in this collection. In this scenario, percutaneous treatment of arrhythmias represents another area of significant progress reflected in this collection. In a study conducted in a single center, Chen et al. (3) reported that catheter ablation is a safe and effective treatment for pediatric atrial tachycardia, with therapeutic success in the short and long term, particularly in children with cardiomyopathy induced by atrial tachycardia. The findings support catheter ablation as a safe and effective therapeutic option for children with drug-resistant atrial arrhythmias and highlight the growing role of interventional electrophysiology in contemporary pediatric cardiology practice.

Precision medicine and genomics emerge as unifying concepts throughout this Research Topic. In a review of genotype-phenotype correlations in pediatric dilated cardiomyopathy, Dai et al. (4) highlighted the marked genetic heterogeneity and distinct clinical trajectories observed in children compared with adults, striking heterogeneity of clinical expression depending on the affected gene. This review demonstrated that the integration of genetic testing with detailed phenotypic characterization is a valuable tool for early diagnosis, refined prognosis, family screening, and individualized follow-up strategies, particularly for positive genotypes.

Inflammatory and systemic diseases with cardiovascular involvement are strongly represented in this Research Topic. Reviews and original studies on Kawasaki disease (KD) provide a comprehensive overview of diagnostic challenges, particularly in incomplete and early-onset presentations. Case series focusing on infants younger than three months underline the heightened risk of coronary artery abnormalities in this vulnerable population. The incomplete clinical presentation of KD in younger children poses an even greater diagnostic challenge (5). The manuscripts reaffirmed that prompt diagnosis, the importance of echocardiography for the evaluation of the coronary arteries, and early therapy with immunoglobulin and aspirin remain the basis for the prevention of long-term coronary complications (5, 6).

The cardiovascular impact of systemic inflammation has gained additional relevance in the post-COVID-19 era. The systematic review addressing multisystem inflammatory syndrome in children synthesizes current evidence on myocardial dysfunction, coronary involvement, and recovery trajectories. The demonstration of persistent subclinical abnormalities in deformation imaging, despite normalization of conventional echocardiographic parameters, raises important questions about long-term surveillance, physical activity counselling, and exercise recommendations in affected children (7). Acknowledging the findings, it is imperative to emphasize the necessity for prospective longitudinal studies to investigate the long-term cardiovascular impact of multisystem inflammatory syndrome in children.

Innovations in the spheres of digital health and artificial intelligence are also represented in this collection by Toba et al. (8). This study developed an artificial intelligence tool to detect abnormalities when analyzing pediatric electrocardiograms, demonstrating diagnostic performance comparable to conventional algorithms, with improved specificity for selected anomalies. As these technologies mature, it will become imperative to undertake rigorous validation procedures and to integrate them into clinical workflows in a judicious manner. This will ensure that these advances augment clinical expertise and promote equitable access to high-quality cardiovascular care (8).

The significance of fetal circulation in postnatal cardiovascular changes, particularly during the neonatal period, is emphasized by Krasic et (9). The authors described the diagnosis of ductus arteriosus thrombosis in a newborn with ductus arteriosus aneurysm (DAA). Thrombosis of the DAA is a well-documented complication, with the potential to result in vascular obstruction or thromboembolic events. This case highlights the importance of early detection and investigation in neonates with echocardiographic findings of intrauterine ductus arteriosus closure, stenosis, or DA closure in the first 12 h of life to prevent life-threatening complications. Collectively, the study reinforces the need for close collaboration among fetal medicine specialists, geneticists, and pediatric cardiologists.

The diagnosis of heart failure in pediatric patients is often challenging, given the possibility of overlapping symptoms with respiratory and/or infectious conditions in children with heart disease. Furthermore, Yin et al. (10) explored the use of biomarkers in the diagnosis of heart failure in children with congenital heart disease. In the present study, the authors conducted an evaluation of the clinical value of *α*-hydroxybutyrate dehydrogenase, cardiac troponin I, and B-type natriuretic peptide in the context of perioperative diagnosis of heart failure in children with congenital heart disease. The study concluded that the levels of these biomarkers are closely correlated with cardiac function, thereby demonstrating high diagnostic efficacy for heart failure.

**Figure 1:**
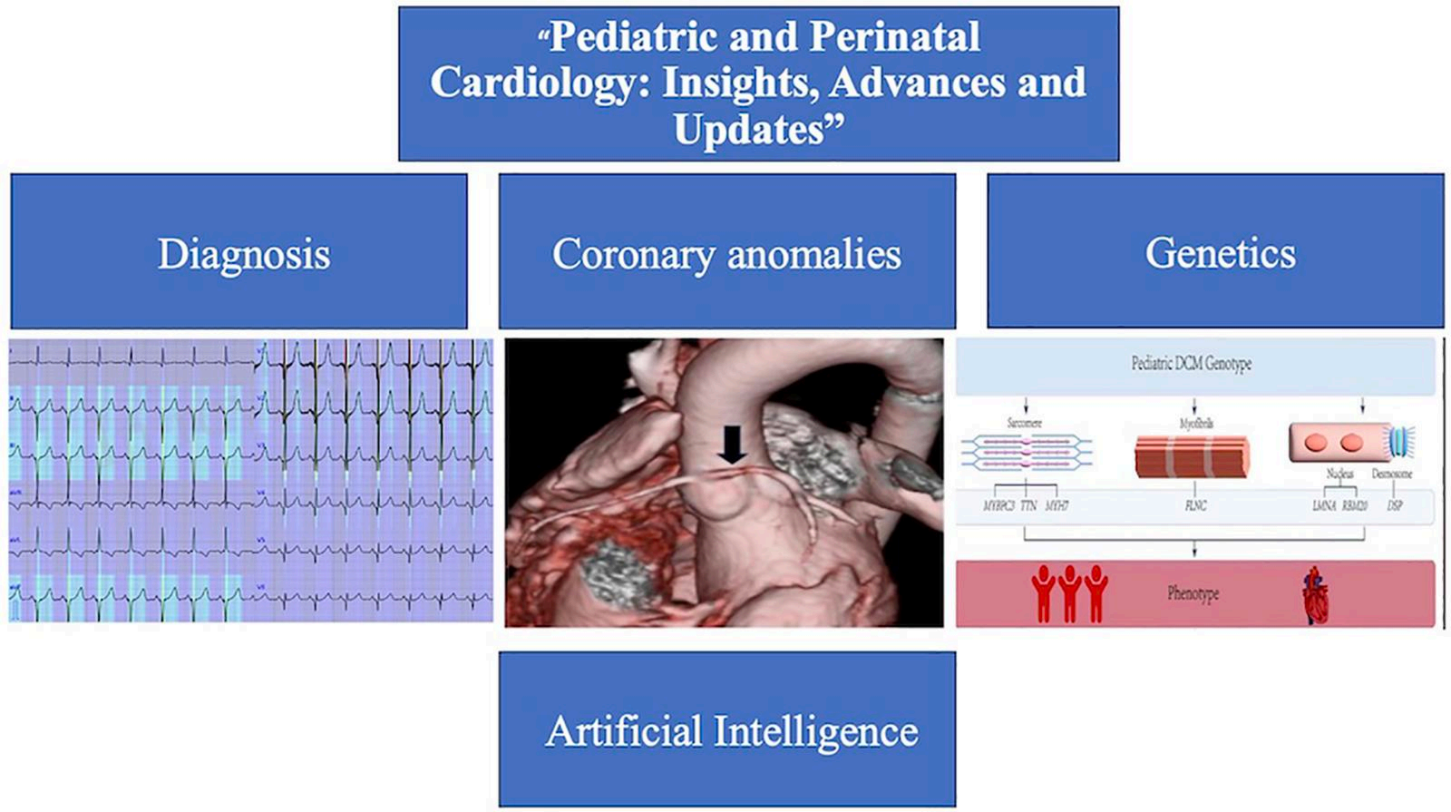
Pediatric and perinatal cardiology: insight's, advances, and updates in diagnostic cardiovascular tools, cardiac percutaneous intervention, cardiogenetic, inflammation research, and artificial intelligence.

In summary, the contributions gathered in ‘Pediatric and Perinatal Cardiology: Insight's, Advances, and Updates’, reflects a dynamic, multidisciplinary field that is increasingly focused on early detection and lifelong cardiovascular health. This Research Topic provided meaningful insights by integrating advances in diagnostic cardiovascular tools, cardiac percutaneous intervention, cardiogenetic, inflammation research, and artificial intelligence (Figure 1). These insights will inform clinical practice and inspire future research aimed at improving outcomes for children with cardiovascular disease worldwide.
